# Coral larval settlement preferences linked to crustose coralline algae with distinct chemical and microbial signatures

**DOI:** 10.1038/s41598-021-94096-6

**Published:** 2021-07-16

**Authors:** Hendrikje Jorissen, Pierre E. Galand, Isabelle Bonnard, Sonora Meiling, Delphine Raviglione, Anne-Leila Meistertzheim, Laetitia Hédouin, Bernard Banaigs, Claude E. Payri, Maggy M. Nugues

**Affiliations:** 1 CRIOBE USR 3278, EPHE-UPVD-CNRS-PSL, 52 Avenue Paul Alduy, 66860 Perpignan Cedex, France; 2 CNRS, Laboratoire d’Ecogéochimie des Environnements Benthiques, LECOB, Sorbonne Université, 66500 Banyuls-sur-Mer, France; 3 Laboratoire d’Excellence « CORAIL», 98729 Papetoai, Moorea French Polynesia; 4 University of the Virgin Islands, St Thomas, 00802-6004 Virgin Islands (U.S.); 5 Plastic@Sea, Observatoire Océanologique de Banyuls, 66650 Banyuls-sur-Mer, France; 6 IRD Nouméa, UMR Entropie, 98848 Nouméa, New Caledonia

**Keywords:** Biochemistry, Chemical biology, Ecology, Microbiology, Ecology, Ocean sciences

## Abstract

The resilience of coral reefs is dependent on the ability of corals to settle after disturbances. While crustose coralline algae (CCA) are considered important substrates for coral settlement, it remains unclear whether coral larvae respond to CCA metabolites and microbial cues when selecting sites for attachment and metamorphosis. This study tested the settlement preferences of an abundant coral species (*Acropora cytherea*) against six different CCA species from three habitats (exposed, subcryptic and cryptic), and compared these preferences with the metabolome and microbiome characterizing the CCA. While all CCA species induced settlement, only one species (*Titanoderma prototypum*) significantly promoted settlement on the CCA surface, rather than on nearby dead coral or plastic surfaces. This species had a very distinct bacterial community and metabolomic fingerprint. Furthermore, coral settlement rates and the CCA microbiome and metabolome were specific to the CCA preferred habitat, suggesting that microbes and/or chemicals serve as environmental indicators for coral larvae. Several amplicon sequence variants and two lipid classes—glycoglycerolipids and betaine lipids—present in *T. prototypum* were identified as potential omic cues influencing coral settlement. These results support that the distinct microbiome and metabolome of *T. prototypum* may promote the settlement and attachment of coral larvae.

## Introduction

Coral reefs are degrading rapidly in response to escalating anthropogenic disturbances^1^. While reef-building corals can recover from minor disturbances, the resilience of coral reef ecosystems after major disturbance events is highly dependent on successful coral recruitment^2^. Corals have a bipartite life history, composed of dispersive larvae, and immobile juvenile and adult stages. This pelago-benthic transition is largely irreversible and therefore coral larvae must recruit onto appropriate benthic surfaces to complete their life cycle and contribute to the recovery of damaged reefs^3^. Coral larvae are capable of sensing fine-scale settlement cues when they actively explore the benthos in the search of a suitable settlement substrate^4^. A variety of physical, biological and chemical cues, ranging from environmental parameters to chemical extracts or bacterial isolates, convey information about the nursery quality of the microhabitat^5,6^. Coral larvae exhibit highly selective responses, and even delay or avoid settlement when facing sub-optimal habitats, highlighting the crucial importance of these cues for coral reef communities^7^.

Crustose coralline algae (CCA) are a group of calcifying red algae commonly found in coral reefs worldwide. They are thought to contribute to reef resilience by acting as highly inductive settlement surfaces for corals^8,9^. However, the specific properties of CCA that induce coral settlement are still highly debated^10,11^. Coral settlement cues associated with CCA are primarily of chemical^8,10,12^ or microbial origin^13–15^. Bacterial culturing experiments have identified several CCA surface-associated bacteria capable of inducing coral settlement when offered as monospecies bacterial films^13,15^. In particular, bacterial isolates within, or affiliated with the genus *Pseudoalteromonas* can induce high levels of coral larval metamorphosis and/or attachment^13–15^. However, its inductive capacity is severely reduced when presented in a mixed species biofilm^16^. To date, the ecological relevance of inductive bacterial isolates in the natural environment remains unclear. Furthermore, these studies are constrained by the limitations of culture-based approaches, which only reveal a small fraction of the microbial community detected by sequencing^17^. Several studies have used culture-independent techniques to examine the bacterial communities associated with CCA. While these studies have shown that CCA species harbour highly diverse and distinct bacterial communities^18–20^, coral settlement has only recently been correlated with the microbiome community composition of CCA^18,20^.

Coral settlement has been induced by contact with non-polar compounds bound in the cell walls of certain CCA species^8,12,21^. A morphogen found within purified cell walls of the CCA *Hydrolithon boergesenii* induced settlement in agaricid corals in the Caribbean^12^. The molecule was associated with, or contained glycosaminoglycan, but was never structurally characterised^12^. More recently, two classes of metabolites found in CCA cell walls (glycoglycerolipids and polysaccharides) have been identified as the main components of the coral settlement-inducing fraction^10^. While both compound classes were detected in natural samples, they have not been successfully characterized and may form bioactive conformational units that cannot be purified^10^. Furthermore, many CCA compounds are produced by epiphytic bacteria and can have a wide array of functions, which range from cytotoxic, antibiotic, antifungal to proteasome inhibitory activities^14,21,22^. However, systematic studies on the metabolomic diversity of CCA species are currently lacking, and information on the microbiome and metabolome of CCA has never been combined to explore their ecological functions.

To help decipher how coral larvae perceive chemical and microbial signals from CCA and their associated bacterial communities, information on the microbiome and metabolome of CCA should be combined and compared with coral settlement preferences. The general goal of this study was thus to test the settlement preferences of the coral *Acropora cytherea* against different CCA species, and to compare these preferences with the CCA metabolome and microbiome characterized by untargeted metabolomics and culture-independent molecular techniques. We used larvae of *A. cytherea* because this genus is an ecologically important coral genus to coral reefs worldwide and its larvae settle and metamorphose in response to CCA^8,9^. Larvae were exposed to six different CCA species, which occupy different habitats (exposed, subcryptic and cryptic) in the coral reefs of Moorea (French Polynesia). We determined settlement preferences on each CCA species in laboratory assays and compared this response to the CCA metabolomic fingerprints and bacterial community composition. The specific aim was to test the following hypotheses: (1) Different CCA species induce different coral settlement preferences. (2) Different CCA species have distinct associated bacterial communities and metabolomic fingerprints. (3) Differences in coral settlement preferences are linked with differences in associated bacterial communities and metabolomic fingerprints. (4) Coral settlement preferences and CCA microbiome and metabolome vary similarly among habitats. We further sought to identify individual bacterial amplicon sequence variants (ASVs) and metabolites possibly involved in coral settlement.

## Results

### Settlement assay

**Figure 1:**
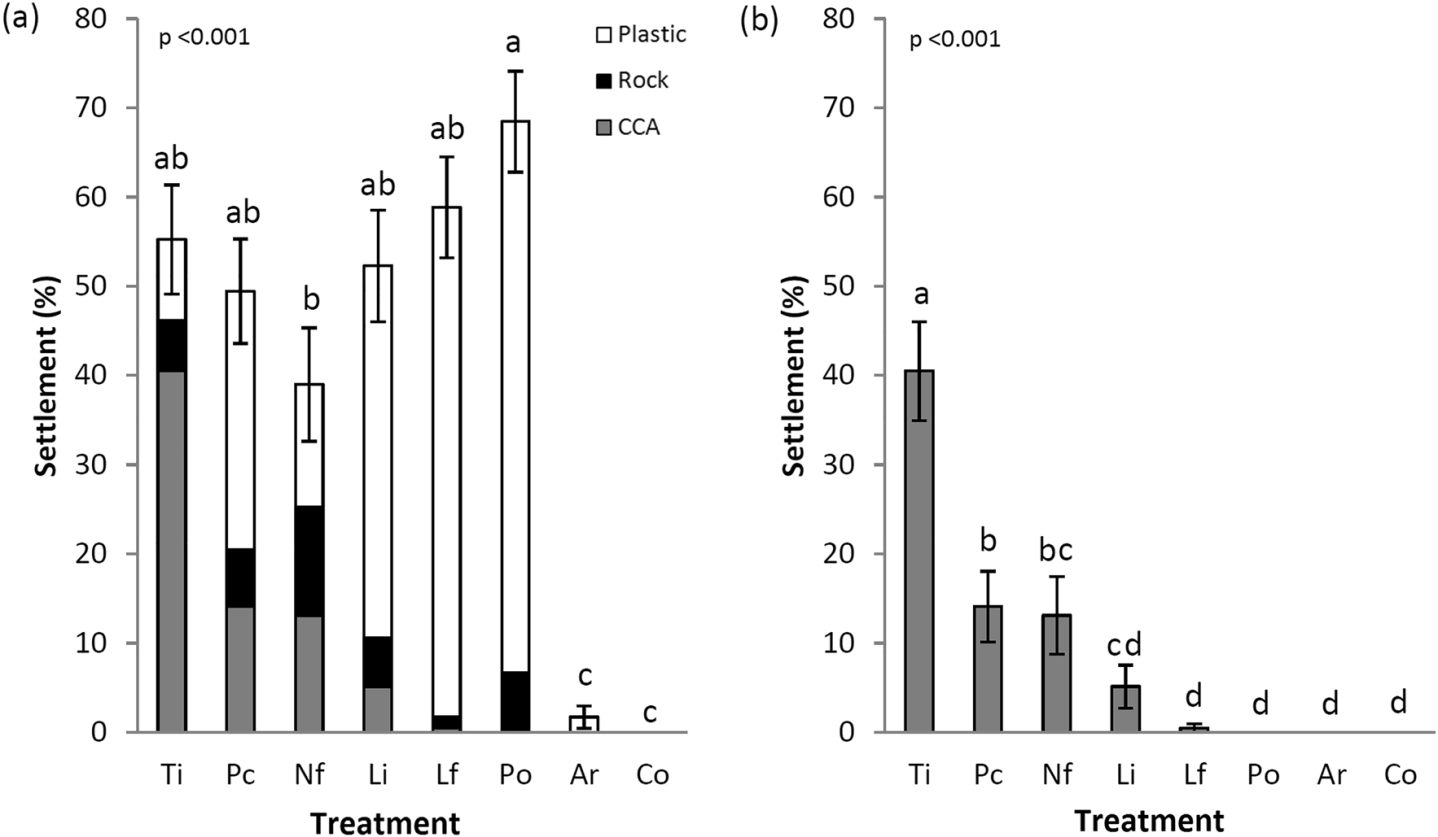
Percentage (mean ± SEM, n = 12 wells) of *Acropora cytherea* larvae that settled in response to the different treatments. (**a**) Total settlement on the different surfaces. (**b**) Settlement on the CCA surface only. Analyzed by Kruskal–Wallis ANOVA on ranks. Letters indicate homogeneous subgroups by Dunnett post hoc tests with Bonferroni correction. Ti, *Titanoderma prototypum*; Nf, *Neogoniolithon fosliei*; Pc, *Paragoniolithon conicum*; Li, *Lithophyllum insipidum*; Lf, *Lithophyllum flavescens*; Po, *Porolithon onkodes*; Ar, aragonite control; Co, FSW control. Cryptic species = Ti; subcryptic species = Pc and Nf; exposed species = Lf, Li and Po.

The larvae of *A. cytherea* had different rates of total settlement in response to the treatments (Fig. 1a; electronic supplementary material, table S1; Kruskal–Wallis H_7_ = 59.85, *p* < 0.001). Rates of total settlement (CCA itself, or underlying limestone rock, or plastic of the well) were highest on *Po. onkodes* (68.4 ± 5.7 SE %), lowest on *N. fosliei* (39.0 ± 6.3%), and intermediate (58.8–49.4%) on the four other CCA species. Hardly any larvae settled in the two controls (aragonite or FSW only). When examining settlement on the CCA surface only, differences in settlement between CCA species were greater (Fig. 1b; electronic supplementary material, table S2; Kruskal–Wallis H_7_ = 67.55, *p* < 0.001). The cryptic CCA species (*T. prototypum*) induced the highest rates of settlement on the surface (40.5 ± 5.6%) compared to all other species, while the exposed species (*L. insipidum*, *L. flavescens* and *Po. onkodes*) induced the lowest rates (< 5.1%).

### Metabolomic fingerprints and richness

**Figure 2:**
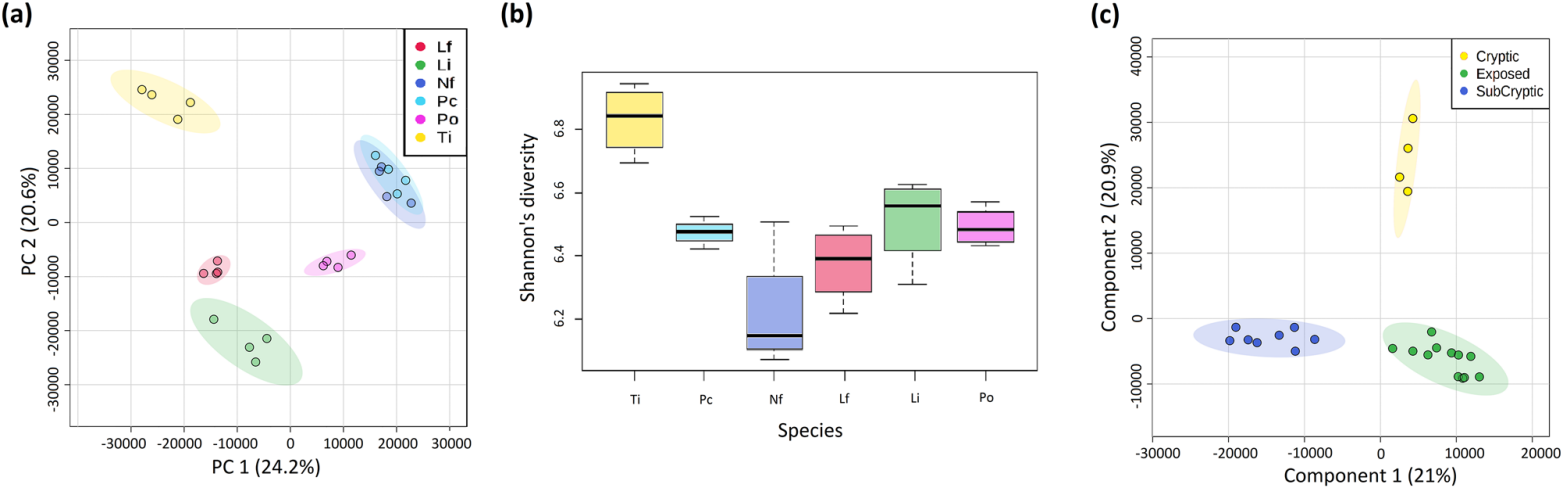
Metabolomic profiles and diversity of the different CCA species. (**a**) Principal component analysis (PCA) of Pareto-scaled normalized ion intensities. (**b**) Box plot showing the diversity (Shannon index) of metabolites. Boxes represent first to last quartile while whiskers represent maximum and minimum values excluding outliers. (**c**) Partial least square discriminant analysis (PLS-DA) model comparing metabolomic profiles among CCA species grouped according to habitats. The amount of variance explained is shown in parenthesis on each PCA and PLS-DA axis. Species abbreviation and habitat as in Fig. 1.

After filtering out potentially redundant ions, a total of 3199 positive mass features were detected in all samples. The composition of the metabolic fingerprints differed significantly between CCA species (PERMANOVA: R = 0.7093, *p* < 0.001). In the PCA ordination (Fig. 2a), the subcryptic species *N. folsei* and *Pa. conicum* clustered together, while the cryptic species *T. prototypum* was clearly distinct from all other species. The exposed species *L. insipidum* and *L. flacescens* were close to each other, while *Po. onkodes* clustered in between the exposed and subcryptic species groups (Fig. 2a; electronic supplementary material, figure S1). The PCA results were supported by pairwise comparisons showing that each CCA species had a significantly different metabolic fingerprints compared with every other species, except from the pair *N. folsei*—*Pa. conicum* (electronic supplementary material, table S3).

Metabolomic diversity differed between CCA species (Fig. 2b; electronic supplementary material, table S4; one way ANOVA: Shannon index: F_5,18_ = 10.71, *p* < 0.001; Number of ions: F_5,18_ = 12.84, *p* < 0.001). The Shannon index and number of ions of *T. prototypum* were higher than those of all other species (electronic supplementary material, tables S5 & S6). *T. prototypum* also had the highest number of union ions and unique union ions of all species (electronic supplementary material, table S4). *Pa. conicum* had the most core ions and unique core ions. *N. folsei* had the fewest core ions and no unique core ions. Metabolome richness and uniqueness were similar among the three exposed CCA species.

Partial least square discriminant analysis (PLS-DA) was used to find the metabolites that contributed most to the discrimination of *T. prototypum*. To increase the discriminative power of the model, the number of groups was reduced using habitat instead of CCA species as factor. The model separated the three habitats (Fig. 2c). Components 1 and 2 explained 21.0% and 20.9% of the total variance, respectively. The robustness of the model was validated (Q2 > 0.8). Using the second component that clearly discriminated the cryptic species *T. prototypum* from the other habitats, we found 15 variables (VIPs) that were present at significantly higher concentrations in *T. prototypum* (electronic supplementary material, table S7). Of these VIPs, ten molecules could be putatively identified (two VIPs were hiding two molecules), four were putatively identified with isomeric uncertainty, and three could not be identified. The metabolites with the highest VIP scores were all glycerolipids from two lipid classes: two monoacylglycerols (MG), seven monogalactosyldiacylglycerols (MGDG), one digalactosyldiacylglycerols (DGDG) from the glycoglycerolipid class, and four diacylglyceryl-N,N,N-trimethylhomoserine (DGTS) or diacylglycerylhydroxymethyl-N,N,N-trimethyl-β-alanine (DGTA) from the betaine lipid class.

### Microbial community composition and richness

**Figure 3:**
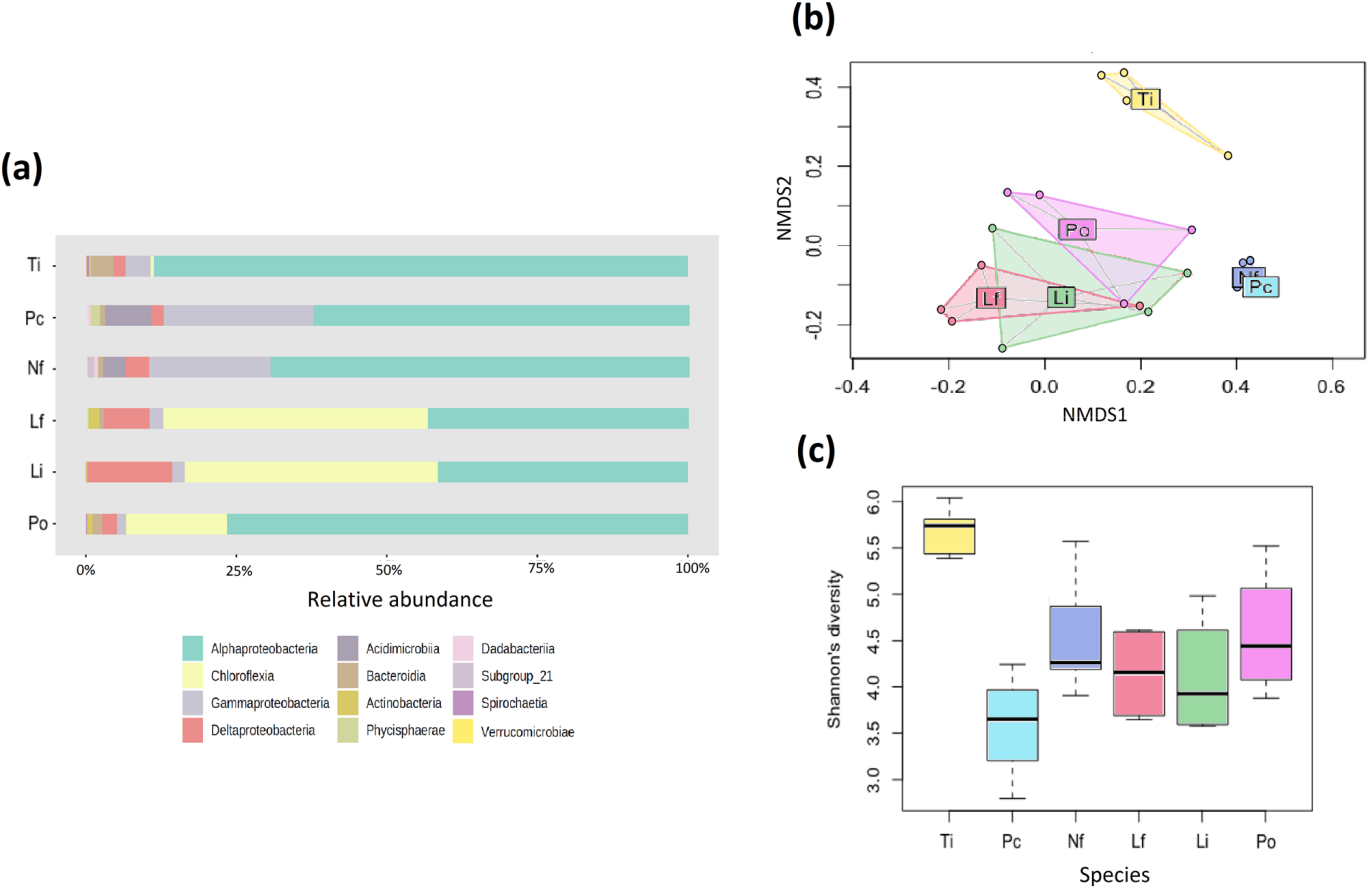
Microbiome profiles and diversity of the different CCA species. (**a**) Relative abundance of ASVs at the class level. (**b**) Nonmetric multidimensional scaling plot based on the Bray–Curtis similarity index. (**c**) Box plot showing the diversity (Shannon index) of ASVs. Boxes represent first to last quartile while whiskers represent maximum and minimum values excluding outliers. Species abbreviation and habitat as in Fig. 1.

A total of 10,251 bacterial 16S rRNA gene sequences composed of 4219 different ASVs were retained after removing eukaryotic sequences (algal chloroplast 16S rRNA), poor quality reads and singletons. At the class level, *Alphaproteobacteria* sequences were relatively more abundant on *T. prototypum* (Fig. 3a), *Gammaproteobacteria* had higher relative abundance on *N. fosliei* and *Pa. conicum*, and *Chloroflexia* were abundant on the exposed CCA species (*L. flavescens, L. insipidium* and *P.onkodes*) and almost or completely absent in the less exposed species*.* At the order level, *Chloroflexales* were abundant in the exposed species, while *Thalassobaculales* and *Alteromodales* were more abundant in the subcryptic species (electronic supplementary material, figure S2). The most frequently encountered orders of bacteria on *T. prototypum* were *Rhodospirillales*, *Thalassobaculales*, *Rhodobacterales*, *Cythophagales* and *Rhizobiales*.

At the ASV level, the composition of the bacterial community differed significantly between CCA species (PERMANOVA: R = 0.5153, *p* < 0.001). The MDS ordination showed that species formed distinct clusters according to their habitats, with the exposed species, the subcryptic species and *T. prototypum* forming three distinct clusters (Fig. 3b). This was supported by pairwise comparisons (electronic supplementary material, table S8). Bacterial diversity (Shannon index) differed between CCA species (Fig. 3c; ANOVA F_5,21_ = 8.13, *p* < 0.001) and was significantly higher in *T. prototypum* than in all other species (electronic supplementary material, table S9).

SIMPER analysis identified 22 ASVs that contributed to over 0.6% of the dissimilarity among CCA species (electronic supplementary material, table S10). The first four ASVs (051, 054, 093, 110) that contributed most to the dissimilarity between CCA species were only found on *T. prototypum* and belonged to *Alphaproteobacteria*. The first two ASVs (051 and 054) belonged to the order of *Thalassobaculales* and were similar (respectively, BLAST: 98.98% similarity and 84% query cover; 98.98% similarity and 83% query cover) to OTUs found earlier associated with *Po. onkodes*^23^ (electronic supplementary material, table S10). The 3rd ASV (ASV093) was strongly similar to “*Roseobacter* sp. S4466” found on macroalgae (BLAST: 99.56% similarity and 97% query cover) and closely related to known algal–bacterial symbionts. Among the remaining ASVs, another four (ASV087, 106, 221, 189) were only present on *T. prototypum* and identified as *Alphaproteobacteria* (electronic supplementary material, table S10). Several ASVs (056, 046, 061, 025, 109, 031, 063) were mainly encountered in the exposed CCA species (*L. insipidium, L. flavescens* and *Po. onkodes*) and in lesser quantities in *T. prototypum*, but never in subcryptic species (*N. fosliei* and *Pa. conicum*). They belonged to the order *Chloroflexales* and closely associated with Chloroflexi bacteria, earlier detected on brown algae, *Cystoseira compressa* (BLAST: 98.62% similarity and 91% query cover)^24^.

### Microbiome-metabolome integration

**Figure 4:**
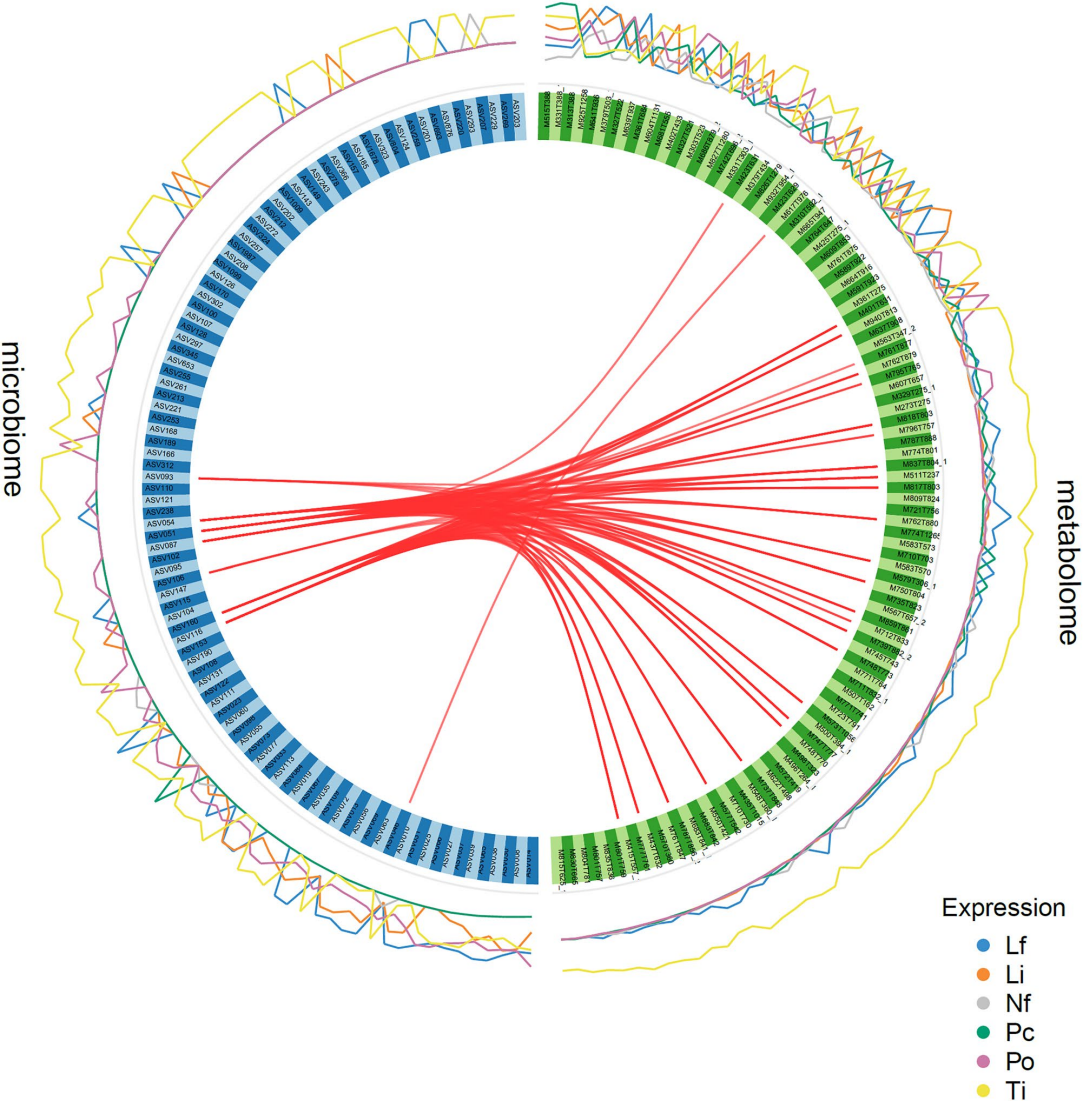
Circos plot depicting the strongest correlations between variables of microbiome (blue blocks) and metabolome (green blocks) data sets (correlation cut-off r ≥ 0.9) and level of relative abundance of each variable for each CCA species. All correlations were positive. Species abbreviation and habitat as in Fig. 1.

A strong correlation between the metabolome and microbiome datasets was observed (r = 0.94 and 0.82 on the first and second component, respectively). The circos plot highlighted strong (r ≥ 0.9) correlations between 8 ASVs and 27 metabolites (Fig. 4). All correlations were positive. The metabolite M617T976 was correlated with the bacterial ASV046 (*Chloroflexales*) earlier found associated to macroalgae (electronic supplementary material, table S10). Both were over represented in *L. flavescens* and *L. insipidum.* All other correlated metabolites and ASVs were over represented in *T. prototypum*. Metabolites M750T804 and M748T770 (electronic supplementary material, table S7), putatively identified as MGDGs, were both correlated with bacterial ASV051, ASV054 and ASV 116 (all three *Thalassobaculales*), ASV093 (*Rhodobacterales*), ASV087 and ASV106 (both *Rhodospirillales*) and ASV160 (unidentified *Alphaproteobacteria*) (electronic supplementary material, table S10). Metabolites M940T813 (putatively identified as a DGDG), M737T848 (putatively identified as a DGTS or DGTA) and unidentified M415T557 (electronic supplementary material, table S7) were all correlated with ASV051, ASV054 and ASV116 (*Thalassobaculales*), ASV087 (*Rhodospirrillales*), ASV160 (unidentified *Alphaproteobacteria*) (electronic supplementary material, table S10).

## Discussion

Our results show that metabolomic and microbiome profiles of CCA differed significantly between species, but that all CCA species induced high (> 54%) rates of total settlement (i.e., settlement on all surfaces—top CCA surface, underlying rock and plastic substrata -). The observation that coral larvae settled in high rates in presence of strongly different microbiome and metabolome suggests that coral settlement can be mediated by several types of microbial biofilm and/or chemicals. The fact that coral settlement, irrespective of settlement substrata, can be induced by numerous CCA species is in agreement with several studies who reported that a wide range of crustose and non-crustose coralline algae species induce high rates of total coral settlement using similar laboratory assays^9,25,26^. However, when considering settlement on the CCA surface only, which represents a substratum that larvae naturally encounter, rates of settlement strongly differed between species. In particular, for *T. prototypum*, a majority of coral larvae settled on its surface rather than on the nearby inert plastic or rock surfaces. Since this species also possessed very distinct associated bacterial communities and metabolomic fingerprints, the direct settlement on the CCA surface could depend on species-specific microbial and/or chemical components.

These apparently contrasting conclusions between total and surface specific settlement could be explained by different larval behavioural responses to CCA species-specific biofilms and/or chemicals. Gómez-Lemos et al.^11^ recently proposed that coral settlement is the result of a synergistic interaction between CCA chemicals and epiphytic microbial biofilms. Specifically, the authors proposed that coral larvae settle in a two-step process: (1) an initial recognition of the epiphytic microbial biofilm by coral larvae mediated by dissolved organic carbon (DOC) produced by CCA, and (2) the metamorphosis of coral larvae in response to specific chemical cues following direct contact with the CCA. In our experiment, in the presence of *T. prototypum*, larvae settled directly on the alga. *T. prototypum* could thus produce positive (chemical and microbial) cues for both steps hypothesized by Gómez-Lemos et al.^11^, ultimately leading to attachment and metamorphosis on the CCA surface itself. In the presence of other CCA species, larvae settled mostly on plastic or rock surfaces and more rarely directly on the CCA surface. These other CCA species could have produced positive microbial cues that initiate the settlement process (i.e., step 1), but negative chemical cues for attachment and metamorphosis (i.e., step 2), driving coral larvae to choose other settlement sites (i.e., rock and/or plastic) on which to attach and metamorphose. These hypotheses are consistent with studies demonstrating coral larval settlement in response to biofilms or isolated bacterial strains only^13,15,16^, as well as in response to chemicals only, or following antibiotic treatments^10,11,15^. Pioneer work has also shown that larvae of several coral species only settle in presence of CCA, but that continued contact is not required since larvae can settle away from CCA surface^27^. Consequently, coral larvae could respond to chemical and microbial stimuli separately or together, each producing different outcomes in terms of overall settlement rates and sites of attachment and metamorphosis. In the natural environment, chemical and microbial stimuli will probably mix and form complex microbial and chemical landscapes shaped by the composition and metabolism of the benthos and hydrodynamic processes^28^. Price^29^ showed that the relative distribution, abundance, and species-species characteristics of preferred CCA species combine to facilitate coral recruitment within a natural environment. In her in situ study, corals recruited more frequently to one species of CCA, *T. prototypum*, and significantly less to other species of CCA, which is consistent with our ex situ results. Our study provides important insights on how these patterns link with the metabolome and microbiome of CCA.

Our study demonstrates that the metabolome and microbiome profiles of CCA differed significantly between species. Species-specific differences in microbiome are in agreement with previous studies on CCA^19,20^ that reported distinct bacterial communities in different species , as well as with research on other marine sessile organisms, such as corals^30^. Metabolomic differences among marine sessile taxa have also been observed previously^31,32^, but never among CCA species. Interestingly, the metabolome and microbiome composition of the different CCA species showed distinct patterns according to the CCA habitats (exposed, subcryptic or cryptic). In particular, different CCA species belonging to the same habitat shared similar metabolomes and microbiomes. These findings indicate that environmental factors, such as light, UV radiation, hydrodynamic regime and herbivory, which differ among habitats, may drive the CCA metabolome and microbiome. Previous research has shown that the relative abundances of lipid classes, as well as their individual components within a class, vary strongly according to physiological state, availability of elemental components (N, P and Si), temperature, salinity, light/dark cycle and growth phase in algal species^33,34^. Differences in metabolomic profiles and diversity following environmental conditions have been reported in several species of corals^35^ and terrestrial plants^36^. Likewise for microbes, environmental parameters, such as temperature and pH, cause microbial shifts in CCA^23,37^. Coral larvae are known to respond to physical parameters, such as light, UV radiation, depth, colour and sound^6^. In particular, light can influence their settlement orientation, with larvae settling in cryptic habitats under high light regimes^38^. The observation that coral larvae settled on a cryptic CCA species with a very distinct microbiome and metabolome suggests that microbial and/or chemical cues could serve as environmental indicators for corals seeking suitable habitat and substrate.

Since we used fragments that were previously used in our settlement assays for microbial analysis, we cannot rule out that some bacteria may have moved from the coral larvae to the CCA during the hours that larvae settled on the CCA. However, the short duration of the experiment (14 h), together with the careful removal of settled larvae, is likely to have minimized any changes in the composition of the CCA natural biofilms due to the addition of coral larvae. It is also important to note that previous research has shown that morphogens can be found in dead CCA and coral skeletons^25^. In the present study, larvae were exposed to both dead skeleton and live CCA surface area (with a dead skeleton to live CCA surface area ratio of approximately 2.2) and could have responded to morphogens located to either or both of those substrates.

The majority of the metabolites that were found at higher concentrations in *T. prototypum* relative to the other CCA species and that could be putatively identified with great certainty were glycoglycerolipids. Glycoglycerolipids have recently gained interest due to their wide range of biological activities^39^. Although these lipids are ubiquitous membrane and cytosolic constituents of all living cells, there is limited knowledge of the biochemical processes underlying their synthesis or plasticity^33,40^. Glycoglycerolipids are present in all photosynthetic life, but they are mainly produced by marine organisms, particularly marine algae and cyanobacteria^39^. They have a wide variety of biological activities such as antibacterial, antiviral, antifungal, antimicroalgal, antiherbivory, allelopathic and antifouling^41,42^. Glycoglycerolipid-containing fractions have been reported as settlement cues for larvae of sea urchins^43^, jellyfish^44^ and corals^10^. Two classes of glycoglycerolipids—monogalactosyl monoacylglycerols (MGMG) and sulfoquinovosyl diacylglycerols (SQDG)—were characterized as main constituents of the coral settlement inducing fractions of *Po. onkodes* in a previous study^10^. Hence, these lipids could indeed play an important role in the recruitment of marine invertebrates. In our study, another class of polar lipids, betaine lipids (BLs) was also present as DGTS and/or DGTA. BLs are regarded as analogous to the phosphatidylcholine in bacteria, fungi, algae, and basal land plants. The balance between BLs and phospholipids has long been understood as an adaptive strategy to deal with periodic phosphorus deficiency in the natural environment. However, in certain algal species, BLs seem to have a distinct role even if there is currently little knowledge on this subject^45^. DGTS are predominant in red algae^46^. DGTA are synthesized from its isomers DGTS; thus, algal species that contain DGTA are expected to contain DGTS^47^. Glycerolipid identification is challenging due to a large number of structural isomers and the formation of different ions with various adducts. In our case, the double bond position of the fatty acids is not known (whatever the glycoglycerolipid) and no characteristic loss of ion could be used to differentiate between the structure of DGTS and DGTA. Only nuclear magnetic resonance (NMR) spectroscopy could characterize DGTS and DGTA. Further research should isolate and exactly identify these metabolites and test them in settlement assays to determine whether they are responsible for settlement induction.

*T. prototypum*, which significantly promoted larvae settlement, had the highest relative abundance of ASVs belonging to *Alphaproteobacteria*. This class of bacteria is known from other CCA microbiome^18–20,23,37,48^, and is also dominant in the early life stages of corals^49^. Eight ASVs that largely contributed to the differences between CCA species belonged to this class and were only present on *T. prototypum*. Among these eight ASVs, the two that most contributed to the dissimilarity between CCA species belonged to the order *Thalassobaculales*, and were closely related to OTUs earlier detected in *Po. onkodes*^23^. However, these two ASVs were not found on *Po. onkodes* in our study. The third most important ASV (093) is probably a typical algal symbiont, which was similar to *“Roseobacter sp. S4466”* (99.56%; BLAST) detected earlier from the surface of marine algae^50^, but its role remains unclear*. Roseobacter* group members inhabit a great variety of marine habitats and niches, and our knowledge of their ecological significance is still limited^51^. They are, however, the first bacteria acquired from seawater by larvae of the Pacific coral *Pocillopora meandrina*^52^. Four other ASVs belonged to the class of *Rhodospirillales*, which was the most abundant class of identified *Alphaproteobacteria* in *T. prototypum*, and, more specifically, to the family of *Rhodospirillaceae*. *Rhodospirillaceae* are important bacteria associated with *Symbiodiniaceae* communities in adult corals globally^53,54^. Several ASVs contributing to dissimilarity between the CCA species were linked to the order of *Chloroflexales* and mainly encountered in the exposed CCA species. These ASVs were closely similar (98.62%) to *Chloroflexi* bacteria that are commonly encountered on canopy-forming macroalgae^24^. The *Chloroflexi* are a phylum primarily composed of gliding, filamentous bacteria possessing a wide diversity of metabolisms and ecological roles, but they are best known as photoheterotrophs^55^. This could explain why they were specifically encountered on the exposed CCA species. Phototrophic *Chloroflexi* bacteria are also important symbionts of several sponges^56^.

In our research, *Pseudoalteromonas* sp. was detected on the surfaces of *L. flavescens*, *N. fosliei* and *T. prototypum*. Isolates of this bacterial strain can induce high levels of coral larval metamorphosis^13–15^. However, its role of natural inducer of coral larval settlement is debated, because its inductive capacity is severely reduced when present in a mixed species biofilm^16^, and the bacterial densities needed to initiate coral larval metamorphosis in laboratory conditions are magnitudes larger than those naturally present on CCA surfaces^10^. In our study, the relative abundances of this taxon were extremely low, consistent with this assessment. Previous studies have suggested that coral larvae could avoid CCA species harbouring bacteria that are closely related to known coral pathogens (*Vibrios* and *Rhodobacteraceae*) or cyanobacteria that are known to produce allelopathic compounds (Oscillatoriales)^19,20^. All our CCA species had low abundances of these taxa and induced high rates of total coral settlement, which is consistent with this suggestion. Interestingly, Siboni et al.^20^ reported a high abundance of *Vibrios* and low coral settlement rates in multiple choice assays on *N. folsei*. These contrasting results suggest that the microbiome may vary strongly within CCA species, maybe in relation with the health status of the host, and that this intraspecific variation may be an important driver of coral settlement preferences. In the same study, coral settlement was positively correlated with ASVs assigned to the *Neptuniibacter*, Methylotrophic Group 3 and *Cellvibrionaceae*^20^. In our study, which amplified the 16S V1-V3 regions, those bacterial taxa were not abundant.

There was a strong correlation between the metabolome and microbiome datasets. Correlations between metabolome and microbial community compositions have been described in several marine sessile organisms, including algae and corals^57^. However, our study is the first to document it for CCA. We were able to identify several strong correlations between ASVs and metabolites that were abundant in *T. prototypum*. Several metabolites that were putatively identified as glycoglycerolipids and betaine lipids were positively correlated with several ASVs, notably those belonging to the orders of *Thalassobaculales*, *Rhodobacterales* and *Rhodospirillales*. Since the inductive role of CCA associated microbial communities on coral settlement may be reliant on the DOC supplied by CCA^11^, further research should combine bacterial isolation and culturing in the presence of primary metabolites derived from CCA with chemical identification to establish whether key bacteria produce key morphogens and to disentangle the complex biochemical communications between CCA, their associated microbial communities and coral larvae. The findings presented here increase our knowledge of the CCA metabolome and microbiome and provide a basis for future research into the role of metabolites and microbial assemblages on coral larval settlement.

## Methods

### Coral collection and larval rearing

Ten *A. cytherea* colonies were collected one day after the full moon in October 2017 from the back reef at ca. 2 m depth on the west coast of the island of Moorea, French Polynesia (17° 33′ 1 4.4″ S 149° 53′ 07.5″ W) and transported to the CRIOBE research station. Colonies were kept in aquaria with flow-through sand filtered water and constant aeration and checked each evening at 19 h. When signs of imminent sperm-egg bundle release were observed, they were isolated in 10 L buckets. Bundles from several colonies (n = 8) were collected and mixed. The mixture was distributed over several small plastic containers (500 ml) containing filtered seawater (0.45 µm, FSW) and left to fertilize for 2 h. After rinsing the sperm and confirming fertilization under a dissecting microscope, embryos were kept on an agitator at slow speed, in a 12:12 light:dark regime and within a temperature range of 26–28 °C. FSW was changed every 12 h and dead planulae were simultaneously removed with the water changes. Five days following spawning, larvae began actively searching the substrate and were considered competent to settle. The settlement assay was conducted 8 days following spawning in the same temperature controlled room in which the larvae were raised. Thus, water temperature in all treatments ranged between 26 and 28 °C.

### CCA collection

Six CCA species were chosen because they are widespread, easy to identify to species level in situ, and differ in habitat characteristics. Three species (*Porolithon onkodes*, *Lithophyllum insipidum* and *Lithophyllum flavescens*, electronic supplementary materials, Figure S3a-c) are most commonly found in areas of the back reef that are exposed to high light and offer easy access to grazers. They are thereafter referred to as exposed species. Two species (*Neogoniolithon fosliei* and *Paragoniolithon conicum,* electronic supplementary materials, Figure S3d-e) are more readily encountered in subcryptic back reef habitats, which are semi-protected habitats on vertical walls or under overhangs. Finally, one species (*Titanoderma prototypum,* electronic supplementary materials, Figure S3f.) was from cryptic habitats that are fully protected habitats inside cavities or consolidated rubbles. Subcryptic and cryptic species are found at lower light intensity and are more protected from grazers. Twelve specimens (approximately 8 cm^2^ of CCA surface) per species were collected from the same back reef site at ca. 2 m depth on the north coast of Moorea (17° 28′ 47.8″ S 149° 50′ 45.3″ W) using hammer and chisel. Only fragments with underlying clean dead skeleton were used. After removing epiphytes, each specimen was cut using pliers to make two fragments: one fragment (approximately 1 cm × 1 cm of CCA surface) immediately used in settlement assays and later preserved for microbial analysis, and one fragment (approximately 4 cm^2^ of CCA surface) immediately stored at − 20 °C and later freeze-dried and stored at − 20 °C for metabolome analysis. The remaining of the specimen was examined using a dissecting microscope and verified at species level using published, anatomically based taxonomic schemes^58,59^.

### Settlement assay

Larvae were haphazardly removed from various larval containers for use in the settlement assay. Treatments included FSW, a 1 cm × 1 cm fragment of aragonite coral frag plugs (Ocean Wonders, IA, USA), and a fragment of one of either six CCA species. CCA fragments consisted of 1 cm^2^ live surface layer of CCA cells upon a section of dead skeleton approximately 3 mm thick, giving a dead skeleton to live CCA surface area ratio of approximately 2.2. The 3 mm thickness was chosen to avoid that fragments break apart, while minimising the presence of dead skeleton laid down by other species. Twelve larvae were added with 12 ml FSW (0.45 µm) into one individual well in a 6-well culture plate (Falcon #353046). One treatment was added to an individual well, and 12 replicate wells were used. Larvae were added at 19:00 and the number of settled larvae that had begun metamorphosis according to the definition of Heyward and Negri^25^ was counted at 9:00 the next day using a dissecting microscope. The substrate on which each larva had settled (i.e., surface of the CCA itself, underlying limestone rock, or plastic of the well) was also recorded. Immediately after scoring, settled larvae were carefully removed using a sterile dissecting needle, and CCA fragments were placed into individual 1.5 ml Eppendorf tubes containing 70% molecular grade EtOH and stored at − 80 °C until DNA extraction. The significance of settlement preferences were tested using non-parametric Kruskal–Wallis one-way ANOVA on ranks followed by Dunnett post hoc tests with Bonferroni correction in R (v.3.3.5).

### Metabolomics sample processing and analyses

Freeze-dried fragments from four randomly selected specimens per CCA species were processed for metabolome analyses (n = 4 replicates per species). A detailed description of metabolomics sample processing, liquid chromatography-mass spectrometry (LC–MS) analysis and data analyses can be found in electronic supplementary materials. In short, the upper (~ 1 mm deep) surface of each fragment was scraped and grinded to a powder (~ 3.0 g per sample). Metabolites were extracted using a biphasic solid–liquid extraction. LC–MS analysis was performed using a UHPLC system interfaced to a QTOF mass spectrometer with an ESI source. LC–MS raw data files were converted to mzXML files with MSConvert, pre-processed and normalized using Workflow4Metabolomics version 3.3 and analysed with MetaboAnalyst 3.0. Species profiles were compared using principle components analysis (PCA). PERMANOVA followed by pairwise comparisons was run to reveal differences in metabolomics fingerprints between CCA species. Shannon index and number of ions were analysed using one way ANOVAs with CCA species as fixed factor, followed by Tukey posthoc tests with Bonferroni correction. Partial least square discriminant analysis (PLS-DA) was used to find the metabolites that contributed most to the discrimination of *T. prototypum*. To increase the discriminative power of the model, the number of groups was reduced using habitat instead of CCA species as factor. Variable Importance in Projection (VIP) was used to summarize the importance of each variable (i.e., metabolite) in driving the separation among habitats. Putative identifications were assessed for the VIPs which were present at significantly higher concentrations in *T. prototypum* (i.e., the cryptic group) relative to the subcryptic and exposed groups.

### DNA extraction, amplicon sequencing and sequence analyses

Fragments from the same four specimens used for metabolome analysis were processed for microbiome analysis (n = 4 replicates per species). A detailed description of DNA extraction, amplicon sequencing and sequence analyses can be found in the electronic supplementary materials. In short, the upper (~ 1 mm deep) surface of each fragment was scraped with a sterilized scalpel. DNA was extracted with a Maxwell 16 MDx Instrument (Promega, WI, USA) following mechanical and chemical lysis, and bacterial 16S rRNA genes were amplified using primers 27F (AGRGTTTGATCMTGGCTCAG) and 519R (GTNTTACNGCGGCKGCTG) and sequenced on the same Miseq Illumina sequencer run (Illumina, CA, United States) to produce a 2 × 300-bp long reads. Sequences were analyzed in R using the Dada2 pipeline and taxonomic assignments of amplicon sequence variants (ASVs) was carried out using the SILVA v132 database. Chimeras and sequences that belonged to algal chloroplast and mitochondria were removed. Sequence data were analyzed using the R package *vegan* after Hellinger transformation and using the STAMP software. Alpha diversity was calculated at the ASV level using the Shannon diversity index and significant differences tested using one way ANOVA with CCA species as fixed factor, followed by Tukey HSD posthoc tests with Bonferroni correction. A non-metric multidimensional scaling ordination (NMDS) was used to visualize community composition between species. PERMANOVA followed by pairwise comparisons was run to reveal differences in bacterial community composition between CCA species at the ASV level. Similarity percentage analysis (SIMPER) was used to determine the ASVs that contributed most to the dissimilarity between CCA species.

### Integration of microbiome and metabolome datasets

Microbiome and metabolomics datasets were integrated using the DIABLO method (Data Integration Analysis for Biomarker discovery using a Latent component method for Omics studies) in the R package *mixOmics*^60^. DIABLO identifies correlated key omics variables in multiple matching datasets measured on the same biological samples^61^. The first 100 ASVs and 100 metabolites separating the different CCA species were used to identify correlations between ASVs and metabolites. Correlations (using a cut-off r ≥ 0.9) were illustrated with a circos plot also showing the level of relative abundance of each variable for each CCA species.

## Supplementary Information

Supplementary Information.

## Data Availability

Raw Illumina sequence reads are available in the NCBI's Sequence Read Archive (SRA) under accession number PRJNA669276. All other data sets are available upon request.
