# Supplementary material for: Coral larval settlement preferences linked to crustose coralline algae with distinct chemical and microbial signatures
Source: Sci Rep. 2021 Jul 16;11:14610. doi: 10.1038/s41598-021-94096-6 (PMC8285400; doi:10.1038/s41598-021-94096-6)
Supplement: Supplementary file 1 — Supplementary Information. [file 41598_2021_94096_MOESM1_ESM.pdf]

Supplementary Information for:

Coral larval settlement preferences linked to crustose coralline algae with distinct chemical and microbial signatures

Hendrikje Jorissen[1], Pierre E. Galand[2], Isabelle Bonnard[1,3], Sonora Meiling[1,4], Delphine Raviglione[1], Anne-Leila Meistertzheim[2,5], Laetitia Hédouin[1,3], Bernard Banaigs[1,3], Claude E. Payri[6], and Maggy M. Nugues[1,3]

[1]EPHE, PSL Research University, UPVD-CNRS, USR3278 CRIOBE, 66860 Perpignan, France; hendrikjejorissen@gmail.com; isabelle.bonnard@univ-perp.fr; sonora.meiling@students.uvi.edu; delphine.raviglione@univ-perp.fr; laetitia.hedouin@criobe.pf; banaigs@univ-perp.fr; maggy.nugues@criobe.pf

[2]Sorbonne Université, CNRS, Laboratoire d'Ecogéochimie des Environnements Benthiques, LECOB, 66500 Banyuls-sur-Mer, France; pierre.galand@obs-banyuls.fr; leila.meistertzheim@gmail.com

[3]Laboratoire d'Excellence « CORAIL», 98729 Papetoai, Moorea, French Polynesia

[4]University of the Virgin Islands, St Thomas, 00802-6004, Virgin Islands

[5]Plastic@Sea, Observatoire Océanologique de Banyuls, 66650 Banyuls-sur-Mer, France

[6]IRD Nouméa, UMR Entropie, 98848 Nouméa, New Caledonia ; claude.payri@ird.fr

Corresponding author: Hendrikje Jorissen, EPHE, PSL Research University, UPVD-CNRS, USR3278 CRIOBE, 66860 Perpignan, France; hendrikjejorissen@gmail.com

Contents:

1. **Supplementary methods**
2. **Supplementary tables**
3. **Supplementary figures**

SUPPLEMENTARY METHODS

Supplementary Methods 1, metabolomics sample processing, liquid chromatography-mass spectrometry (LC-MS) analysis and data analyses:

Freeze-dried fragments from four randomly selected specimens per CCA species were processed for metabolome analyses (n = 4 replicates). The upper (~1mm deep) surface of each fragment was scraped and grinded to a powder (approximately 3.0 g per sample) into separate 1 mL glass hemolysis tubes using a file. A 2:1:4 solvent mixture of water, methanol and methyl tert-butyl ether was added to perform a biphasic solid-liquid extraction. The supernatant was separated in an organic phase and a hydroalcoholic phase, after which solvents were evaporated using a Genevac™ centrifugal concentrator. The organic phase was resolubilised in methanol at 1 mg/mL before LC-MS analysis. LC-MS analysis was performed using a UHPLC system (Vanquish Thermo Scientific, MA, USA) interfaced to a QTOF mass spectrometer (MaXis, Brucker, Daltonics, MA, USA) with an ESI source. The chromatographic separation was carried out on a Luna Omega 1.6 µm Polar C18 column (Phenomenex, CA, USA) with a linear gradient of solvents (H_2O + 1 ‰ formic acid and MeOH/Isopropanol (50/50) + 1 ‰ formic acid). MS spectra were acquired in positive ion mode with full scan MS window of 200-1600 *m/z,* capillary voltage of 3500 V, ion source temperature 200 °C, nebuliser gas pressure 35 psi, dry gas flow 8 L/min, spectral rate of 3 Hz for MS1 and 10 Hz for MS2. MS/MS fragmentation of the 7 most intense selected ions per spectrum was performed using ramped collision-induced dissociation energy, ranging from 20 eV to 50 eV. Samples were analysed in a blocked design with quality

control samples and methanol blanks to minimize any potential bias due to instrumental drift. Molecular formula and fragmentation spectra were confirmed by analysis on a QEXactive plus Orbitrap mass spectrometer (ThermoScientific, MA, USA). The chromatographic separation was carried out as above. The mass spectrometer analyzer parameters were set as follows: sheath gas flow rate: 35 a.u (arbitrary units), aux gas flow rate: 10 a.u, sweep gas flow rate: 0 a.u, capillary temperature: 320 °C, S-lens RF level: 50, aux gas temperature: 200 °C, scan range: 100-1500 m/z, spray voltage: 3,5 kV. Full MS resolution: 70000, Data Dependant MS/MS resolution: 17500, top 5, isolation window: 1,5 m/z, NCE: 20, 30 et 40 eV.

All data files were converted to mzXML files with MSConvert (ProteoWizard 3.0). Pre-processing, normalization and quality checks were performed using Workflow4metabolomics version 3.3 [1]. Processed data were analysed with MetaboAnalyst 3.0 after having been Pareto scaled (i.e., mean-centered and divided by the square root of the standard deviation of each variable) to provide equivalent weight among variables [2]. Species profiles were compared using principle components analysis (PCA). PERMANOVA followed by pairwise comparisons was run to reveal differences in metabolomics fingerprints between CCA species. Shannon index and number of ions were analysed using one way ANOVAs with CCA species as fixed factor, followed by Tukey posthoc tests. To compare metabolomic richness, ions were classified as ‘union’ if present in at least one sample of a given species and classified as ‘core’ if present in all samples of that species. Union and core ions were classified as ‘unique’ to a species if they were absent in all samples outside that species.

Partial least square discriminant analysis (PLS-DA) was used to find the metabolites that contributed most to the discrimination of *T. prototypum*. To increase the discriminative power of the model, the number of groups was reduced using habitat instead of CCA species as factor. Significance of PLS-DA model was assessed with permutation tests (consisting of 1000

permutations) and leave one out cross-validation (LOOCV). Robustness of PLS-DA model was validated by calculating Q2. Variable Importance in Projection (VIP) was used to summarize the importance of each variable (i.e., metabolite) in driving the separation among habitats. Using the exact mass, the molecular formulas were estimated and putative identifications were assessed for the VIPs which were present at significantly higher concentrations in *T. prototypum* (i.e., the cryptic group) relative to the subcryptic and exposed groups. Identifications were strengthened by fragmentation spectra issued from MS/MS analyses and according to literature and databases, including m/z cloud, KEGG, LIPID MAPS, Metlin and MarinLit.

Supplementary Methods 2, DNA extraction, amplicon sequencing and sequence analyses: Fragments from the same four specimens used for metabolome analysis were processed for microbiome analysis (n = 4 replicates). DNA extraction followed the protocol of Meistertzheim et al. [3]. Briefly, this protocol starts with a mechanical lysis using a FastPrep Instrument (MP Biomedical, CA, USA) with Y Matrix tubes, followed by a chemical lysis phase by incubation for 1h at 56°C with added proteinase K. DNA was extracted using Maxwell 16 MDx Instrument with the Maxwell Blood DNA Purification Kit LEV (Promega, WI, USA). DNA concentrations were verified using a Victor Spectrofluorimeter (PerkinElmer Inc, Netherlands) with PicoGreen® dsDNA quantitation assays (Thermo Fisher Scientific, MA, USA).

The V1-V3 regions of bacterial 16S rRNA genes were amplified by PCR using bacteria specific primers 27F (AGRGTTTGATCMTGGCTCAG) and 519R (GTNTTACNGCGGCKGCTG) with the barcode on the forward primer. Based on their DNA concentrations, samples were pooled in equal proportions and purified using calibrated Agencourt AMPure XP (Beckman Coulter, CA, USA). Pooled and purified PCR products were then used to prepare a DNA library following the Illimina TruSeq DNA protocol. Using Miseqreagent kit V3 (Illumina), samples were sequenced

on the same Miseq Illumina sequencer run (Illumina, CA, United States) to produce a 2 x 300-bp long reads sequenced at the CGEB-Integrated Microbiome Resource (IMR, cgeb-imr.ca), Dalhousie University, Canada.

Sequences were analysed using the standard Dada2 pipeline in R [4]. The R1 and R2 reads were filtered, trimmed, and merged to create an amplicon sequence variant (ASV) table. This is a higher resolution analogue to the traditional OTU table, which records the number of times each exact amplicon sequence variant is observed in each sample. Chimeras were removed and taxonomy was assigned using the SILVA v132 database [5]. The taxonomic affiliation of ASVs of interest was further verified against sequences from the NCBI database using BLAST. Sequences that belonged to algal chloroplast and mitochondria were removed.

Sequence data were analysed using the R package vegan after Hellinger transformation [6] and using the STAMP software [7]. Alpha diversity was calculated at the ASV level using the Shannon diversity index and analysed using one way ANOVA with CCA species as fixed factor, followed by TukeyHSD posthoc tests with Bonferroni correction. A non-metric multidimensional scaling ordination (NMDS), based on the Bray-Curtis similarity, was used to visualize community composition between species. PERMANOVA followed by pairwise comparisons was run to reveal differences in bacterial community composition between CCA species at the ASV level. Similarity percentage analysis (SIMPER) was used to determine the ASVs that contributed most to the dissimilarity between CCA species.

Supporting References:

1. Giacomoni F, Le Corguillé G, Monsoor M, Landi M, Pericard P, Pétéra M, et al. Workflow4Metabolomics: a collaborative research infrastructure for computational metabolomics. *Bioinforma Oxf Engl* 2015; **31**: 1493–1495.

2. Xia J, Sinelnikov IV, Han B, Wishart DS. MetaboAnalyst 3.0--making metabolomics more meaningful. *Nucleic Acids Res* 2015; **43**: W251-257.
3. Meistertzheim A-L, Nugues MM, Quéré G, Galand PE. Pathobiomes differ between two diseases affecting reef building coralline algae. *Front Microbiol* 2017; **8**.
4. Callahan BJ, McMurdie PJ, Rosen MJ, Han AW, Johnson AJA, Holmes SP. DADA2: High-resolution sample inference from Illumina amplicon data. *Nat Methods* 2016; **13**: 581–583.
5. Quast C, Pruesse E, Yilmaz P, Gerken J, Schweer T, Yarza P, et al. The SILVA ribosomal RNA gene database project: improved data processing and web-based tools. *Nucleic Acids Res* 2013; **41**: D590–D596.
6. Oksanen J, Blanchet FG, Kindt R, Legendre P, Minchin PR, O'Hara RB, et al. vegan: Community ecology package. 2012.
7. Parks DH, Tyson GW, Hugenholtz P, Beiko RG. STAMP: statistical analysis of taxonomic and functional profiles. *Bioinformatics* 2014; **30**: 3123–3124.

SUPPLEMENTARY TABLES

Supplementary table S1: Results of Kruskal-Wallis ANOVA on ranks and Dunnett pairwise tests with Bonferroni correction comparing total settlement of *Acropora cytherea* larvae among treatments. Significant p values ($P < 0.05$) are in bold. Ti = *Titanoderma prototypum*, Nf = *Neogoniolithon fosliei*, Pc = *Paragoniolithon conicum*, Li = *Lithophyllum insipidum*, Lf = *Lithophyllum flavescens*, Po = *Porolithon onkodes*, Ar = aragonite control, Co = FSW control.

Source	df	F	p	Pairwise	p
Treatment	7	59.85	**<0.001**	Ti vs Pc	0.762
				Ti vs Nf	0.289
				Ti vs Li	0.851
				Ti vs Lf	0.822
				Ti vs Po	0.261
				Ti vs Ar	**<0.001**
				Ti vs Co	**<0.001**
				Pc vs Nf	0.488
				Pc vs Li	0.854
				Pc vs Lf	0.547
				Pc vs Po	0.177
				Pc vs Ar	**<0.001**
				Pc vs Co	**<0.001**
				Nf vs Li	0.359
				Nf vs Lf	0.169
				Nf vs Po	**0.019**
				Nf vs Ar	**0.012**
				Nf vs Co	**0.007**
				Li vs Lf	0.728
				Li vs Po	0.261
				Li vs Ar	**<0.001**
				Li vs Co	**<0.001**
				Lf vs Po	0.504
				Lf vs Ar	**<0.001**
				Lf vs Co	**<0.001**
				Po vs Ar	**<0.001**
				Po vs Co	**<0.001**
				Ar vs Co	0.873

Supplementary table S2: Results of Kruskal-Wallis ANOVA on ranks and Dunnett pairwise tests with Bonferroni correction comparing the settlement of *Acropora cytherea* larvae on CCA surface among treatments. Significant p values ($P < 0.05$) are in bold. Species abbreviation as in table S1.

Source	df	F	p	Pairwise	p
Treatment	7	67.55	**<0.001**	Ti vs Pc	**0.047**
				Ti vs Nf	**0.039**
				Ti vs Li	**<0.001**
				Ti vs Lf	**<0.001**
				Ti vs Po	**<0.001**
				Ti vs Ar	**<0.001**
				Ti vs Co	**<0.001**
				Pc vs Nf	0.951
				Pc vs Li	**0.049**
				Pc vs Lf	**0.002**
				Pc vs Po	**0.001**
				Pc vs Ar	**0.001**
				Pc vs Co	**0.001**
				Nf vs Li	0.088
				Nf vs Lf	**0.004**
				Nf vs Po	**0.002**
				Nf vs Ar	**0.002**
				Nf vs Co	**0.002**
				Li vs Lf	0.208
				Li vs Po	0.146
				Li vs Ar	0.131
				Li vs Co	0.138
				Lf vs Po	0.945
				Lf vs Ar	0.869
				Lf vs Co	0.906
				Po vs Ar	1.000
				Po vs Co	1.000
				Ar vs Co	1.000

Supplementary table S3: Pairwise comparisons with Bonferroni correction of metabolomic fingerprints associated with the different CCA species following PERMANOVA. P-values are given above the diagonal and R values are given below the diagonal. Significant p values ($P < 0.05$) are in bold. Species abbreviations as in table S1

	p-values					
	Ti	Pc	Nf	Lf	Li	Po
R-values						
Ti		**0.036**	**0.033**	**0.045**	**0.042**	**0.048**
Pc	0.526		0.390	**0.036**	**0.039**	**0.027**
Nf	0.425	0.249		**0.041**	**0.036**	**0.045**
Lf	0.322	0.521	0.382		**0.033**	**0.024**
Li	0.332	0.501	0.362	0.217		**0.022**
Po	0.323	0.437	0.318	0.291	0.217	

Supplementary table S4: Comparison of richness and uniqueness of the metabolomes of the different CCA species.

Species	Mean ions/sample ± SEM	Core ions	Unique Core Ions	Union ions	Unique Union Ions
Ti	931.50 ± 50.45	242	11	1940	629
Pc	634 ± 19.43	365	53	963	215
Nf	509.75 ± 54.04	87	0	1083	204
Li	680.5 ± 45.76	129	6	1495	284
Lf	589.25 ± 34.22	121	5	1276	152
Po	661 ± 20.67	160	0	1386	213

Supplementary table S5: One-way ANOVA and Tukey HSD pairwise tests with Bonferroni correction comparing metabolomic diversity (Shannon index) among CCA species. Significant p values ($P < 0.05$) are in bold. Species abbreviation as in table S1.

Source	df	F	p	Pairwise	p
Species	5	10.71	**<0.001**	Ti vs Pc	**0.004**
				Ti vs Nf	**<0.001**
				Ti vs Li	**0.021**
				Ti vs Lf	**<0.001**
				Ti vs Po	**0.011**
				Pc vs Nf	0.137
				Pc vs Li	0.974
				Pc vs Lf	0.947
				Pc vs Po	0.996
				Nf vs Li	**0.003**
				Nf vs Lf	0.510
				Nf vs Po	0.056
				Li vs Lf	0.598
				Li vs Po	0.999
				Lf vs Po	0.753

Supplementary table S6: One-way ANOVA and Tukey HSD pairwise tests with Bonferroni correction comparing the number of ions among CCA species. Significant p values ($P < 0.05$) are in bold. Species abbreviation as in table S1.

Source	df	F	p	Pairwise	p
Species	5	12.84	**<0.001**	Ti vs Pc	**<0.001**
				Ti vs Nf	**<0.001**
				Ti vs Li	**0.004**
				Ti vs Lf	**<0.001**
				Ti vs Po	**0.001**
				Pc vs Nf	0.283
				Pc vs Li	0.959
				Pc vs Lf	0.965
				Pc vs Po	0.996
				Nf vs Li	0.067
				Nf vs Lf	0.721
				Nf vs Po	0.128
				Li vs Lf	0.598
				Li vs Po	0.999
				Lf vs Po	0.796

Supplementary table S7: Mass measurements, molecular formulas and putative identification of the 15 VIPs showing a higher abundance in *T. prototypum* relative to exposed and subcryptic species groups. VIPs are ranked according to their VIP scores. Abbreviations: *m/z* = mass-to-charge ratio, A1 = $[M+Na-FA1]^+$ or $[M+H-FA1]^+$, A2 =$[M+Na-FA2]^+$ or $[M+H-FA2]^+$, FA = Fatty Acids, PI = putatively identified, PII = putatively identified with isomeric uncertainty, TM = two molecules under one peak, NF = not found.

VIPs	parent ion form	*m/z* measured	Error ppm	Molecular formula	A1 *m/z*	A2 *m/z*	FA1	FA2	Putative ID	Compound
M425T275		425.1931							NF	
M329T275	$[M+H]^+$	329.2056	0.19	$C_{19}H_{36}O_4$			16:1*		PI	MG(16:1/0:0/0:0)**
M331T303	$[M+H]^+$	331.2843	0.04	$C_{19}H_{38}O_4$			16:0*		PI	MG(16:0/0:0/0:0)**
M579T306		579.2642							NF	
M415T557		415.3931							NF	
M745T743	$[M+Na]^+$	745.4858	-0.43	$C_{41}H_{70}O_{10}$	493.277	493.277	16:2	16:2	PI	MGDG(16:2/16:2)
M771T764	$[M+Na]^+$	771.5016	-0.22	$C_{43}H_{72}O_{10}$	493.277	519.292	18:3	16:2	TM	MGDG(18:3/16:2)
					491.261	521.308	18:2	16:3	TM	MGDG(18:2/16:3)
M748T770	$[M+Na]^+$	747.5014	0.49	$C_{41}H_{72}O_{10}$	495.292	493.277	16:2	16:1	PI	MGDG(16:2/16:1)
M774T801	$[M+Na]^+$	773.5170	-0.54	$C_{43}H_{74}O_{10}$	495.293	519.291	18:3	16:1	TM	MGDG(18:3/16:1)
					493.277	521.308	18:2	16:2	TM	MGDG(18:2/16:2)
M750T804	$[M+Na]^+$	749.5173	-0.16	$C_{41}H_{78}O_{10}$	495.293	495.293	16:1	16:1	PI	MGDG(16:1/16:1)
M940T813	$[M+Na]^+$	939.6014	-0.15	$C_{49}H_{88}O_{15}$	659.361	683.361	18:2	16:0	PI	DGDG(18:2/16:0)
M735T825	$[M+H]^+$	734.5931	0.23	$C_{44}H_{80}NO_7$	478.353	456.387	16:0*	18:2*	PII	DGTS/DGTA(16:0/18:2)**
M711T832	$[M+H]^+$	710.5934	0.66	$C_{42}H_{80}NO_7$	428.337	482.384	14:0*	18:1*	PII	DGTS/DGTA(14:0/18:1)**
M835T836	$[M+H]^+$	834.6236	-0.99	$C_{52}H_{84}NO_7$	504.368	530.384	20:4*	22:5*	PII	DGTS/DGTA(20:4/22:5)**
M737T848	$[M+H]^+$	736.6086	0.03	$C_{44}H_{82}NO_7$	480.368	456.368	16:0*	18:3*	PII	DGTS/DGTA(16:0/18:3)**

* = Uncertainty about the location of fatty acids in the formula (position *sn*-1 or *sn*-2 on the glycerol)
** = the name of the lipid depends of the FA position

Supplementary table S8: Pairwise comparisons with Bonferroni correction of bacterial communities associated with the different CCA species following PERMANOVA. P-values are given above the diagonal. (p-values < 0.05 are indicated in bold) and R values are given below the diagonal. Species abbreviations as in table S1.

	p-values					
	Ti	Pc	Nf	Lf	Li	Po
R-values						
Ti		**0.045**	**0.046**	**0.030**	**0.045**	**0.030**
Pc	0.467		0.755	**0.039**	**0.042**	**0.041**
Nf	0.437	0.330		**0.017**	**0.040**	**0.025**
Lf	0.522	0.489	0.458		1.000	0.285
Li	0.466	0.415	0.383	0.326		1.000
Po	0.416	0.422	0.387	0.393	0.342	

Supplementary table S9: One-way ANOVA and Tukey HSD pairwise tests with Bonferroni correction comparing microbiome diversity (Shannon index) among CCA species. Significant p values (P < 0.05) are in bold. Species abbreviation as in table S1.

Source	df	F	p	Pairwaise	p
Species	5	8.13	**<0.001**	Ti vs Pc	**<0.001**
				Ti vs Nf	**0.045**
				Ti vs Li	**0.003**
				Ti vs Lf	**0.005**
				Ti vs Po	**0.049**
				Pc vs Nf	0.092
				Pc vs Li	0.796
				Pc vs Lf	0.722
				Pc vs Po	0.101
				Nf vs Li	0.677
				Nf vs Lf	0.759
				Nf vs Po	0.999
				Li vs Lf	0.999
				Li vs Po	0.665
				Lf vs Po	0.744

Supplementary table S10: SIMPER (similarity percentage) analysis showing the ASVs (amplicon sequence variant s) contributing >0.6% to the dissimilarity among the different CCA species. Species abbreviations as in table S1.

ASV	% contr.	Mean % abundance (# samples containing ASV)												SILVA		NCBI BLAST		
	to dissim.	Ti		Pc		Nf		Lf		Li		Po		Order	Family	Closest match source	% ID	Accession #
ASV051	0.9688	1.27	(4)	0	(0)	0	(0)	0	(0)	0	(0)	0	(0)	Thalassobaculales	NA	CCA-associated	98.98	JQ178628.1
ASV054	0.9552	1.33	(4)	0	(0)	0	(0)	0	(0)	0	(0)	0	(0)	Thalassobaculales	NA	CCA-associated	98.98	JQ178628.1
ASV093	0.9425	0.87	(4)	0	(0)	0	(0)	0	(0)	0	(0)	0	(0)	Rhodobacterales	Rhodobacteraceae	macro-algae associated	99.56	FJ460048.1
ASV110	0.8959	0.85	(4)	0	(0)	0	(0)	0	(0)	0	(0)	0	(0)	Rhodospirillales	Rhodospirillaceae	coral-associated (Montastrea)	99.15	JQ516442.1
ASV122	0.8480	0	(0)	0	(0)	0	(0)	2.79	(3)	0.28	(2)	0.12	(1)	Alphaproteobacteria (class)	NA	coral-associated (Acropora)	94.38	GU118209.1
ASV087	0.8440	0.86	(4)	0	(0)	0	(0)	0	(0)	0	(0)	0	(0)	Rhodospirillales	Rhodospirillaceae	coral-associated (Montastrea)	99.35	GU118711.1
ASV098	0.8368	0.91	(4)	3.85	(3)	0	(0)	0	(0)	0	(0)	0	(0)	Rhodospirillales	Rhodospirillaceae	coral-associated (Diplora)	97.83	GU118265.1
ASV106	0.7900	0.89	(4)	0	(0)	0	(0)	0	(0)	0	(0)	0	(0)	Rhodospirillales	Rhodospirillaceae	coral-associated (Montastrea)	99.35	GU118711.1
ASV221	0.7525	0.52	(3)	0	(0)	0	(0)	0	(0)	0	(0)	0	(0)	Rhodospirillales	Rhodospirillaceae	CCA-associated	99.5	JQ179217.1
ASV189	0.7451	0.54	(3)	0	(0)	0	(0)	0	(0)	0	(0)	0	(0)	Alphaproteobacteria (class)	NA	coral-associated (Montastrea)	96.36	GU118868.1
ASV160	0.7388	0.55	(3)	0	(0)	0	(0)	0	(0)	0	(0)	0.06	(1)	Alphaproteobacteria (class)	NA	coral-associated (Acropora)	99.78	GU117977.1
ASV116	0.7145	0.71	(3)	0	(0)	0	(0)	0	(0)	0	(0)	0.11	(1)	Thalassobaculales	NA	coral-associated (Diplora)	99.78	GU117977.1
ASV056	0.6887	0	(0)	0	(0)	0	(0)	2.94	(3)	2.14	(2)	0.55	(2)	Chloroflexales	NA	macro-algae associated	98.62	KU689582.1
ASV046	0.6815	0	(0)	0	(0)	0	(0)	3.14	(3)	2.14	(2)	1.02	(2)	Chloroflexales	NA	macro-algae associated	98.62	KU689582.1
ASV014	0.6790	1.44	(4)	0	(0)	0	(0)	2.36	(3)	0.84	(2)	2.84	(3)	Rhodospirillales	Rhodospirillaceae	CCA-associated	99.75	JQ178787.1
ASV061	0.6606	0.05	(1)	0	(0)	0	(0)	2.25	(3)	1.44	(2)	0.57	(2)	Chloroflexales	Chloroflexaceae	macro-algae associated	98.62	KU689582.1
ASV025	0.6571	0.07	(1)	0	(0)	0	(0)	3.50	(3)	2.10	(2)	1.48	(2)	Chloroflexales	Chloroflexaceae	macro-algae associated	98.62	KU689582.1
ASV109	0.6565	0	(0)	0	(0)	0	(0)	2.37	(3)	1.37	(2)	0.35	(2)	Chloroflexales	NA	macro-algae associated	98.62	KU689582.1
ASV031	0.6418	0.21	(2)	0	(0)	0	(0)	3.39	(3)	2.28	(2)	0.86	(2)	Chloroflexales	NA	macro-algae	98.62	KU689582.1
ASV066	0.6219	0.49	(4)	0	(0)	0	(0)	1.32	(3)	0	(0)	1.13	(2)	Rhodospirillales	Rhodospirillaceae	CCA-associated	100	JQ178656.1
ASV108	0.6175	0.77	(4)	0	(0)	0.27	(3)	0	(0)	0	(0)	0	(0)	Alphaproteobacteria (class)	NA	coral-associated (Montastrea)	96.15	GU118868.1
ASV063	0.6045	0.13	(2)	0	(0)	0	(0)	2.61	(3)	1.41	(2)	0.59	(2)	Chloroflexales	NA	macro-algae	98.62	KU689582.1

Closest order match is based on percent similarity in the SILVA database. Closest match source is based on BLAST search of the NCBI database.

SUPPLEMENTARY FIGURES

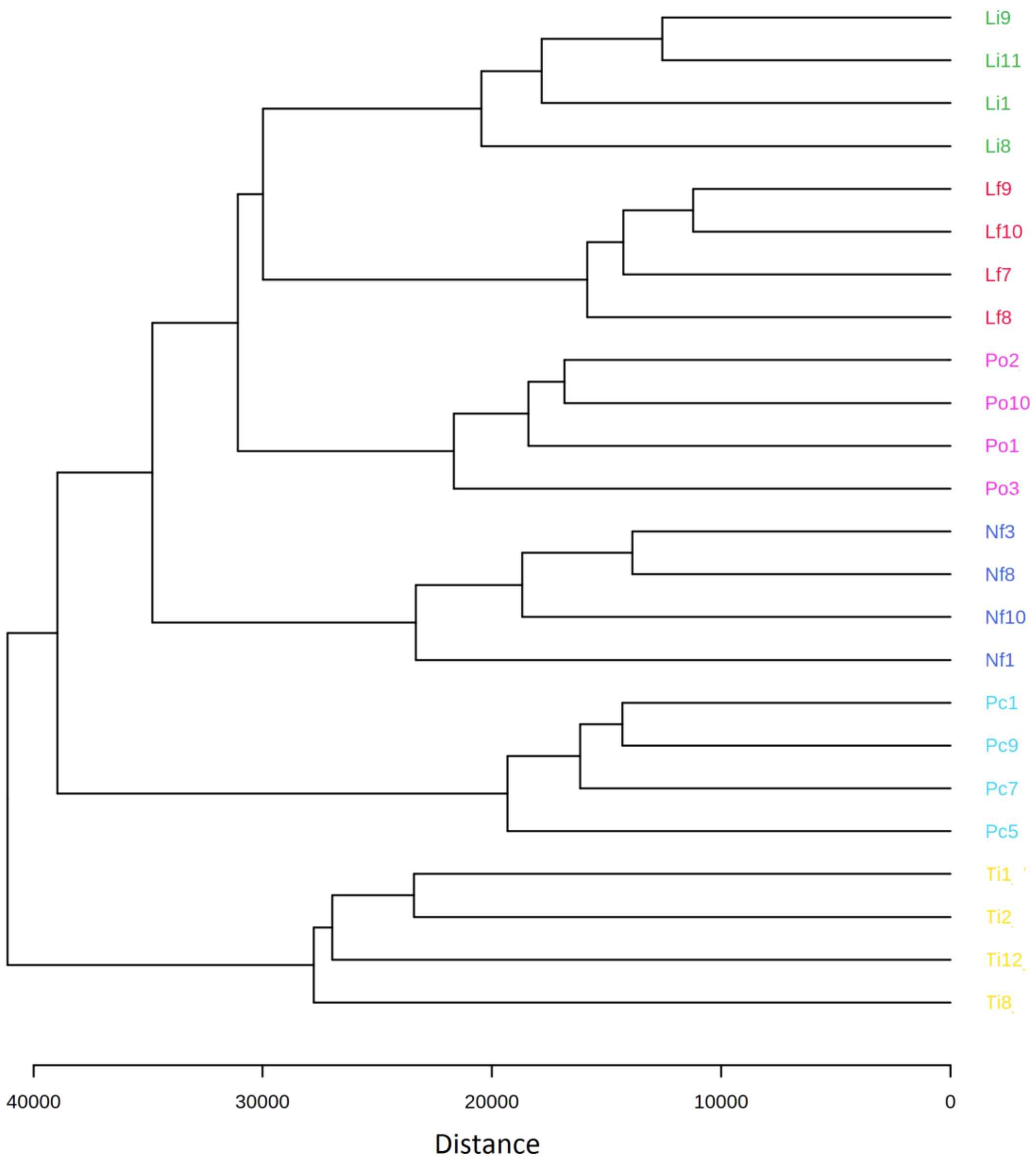

Supplementary figure S1: Hierarchical clustering analysis of normalized ion intensities for the different CCA species. Ti = *Titanoderma prototypum*, Nf = *Neogoniolithon fosliei*, Pc = *Paragoniolithon conicum*, Li = *Lithophyllum insipidum*, Lf = *Lithophyllum flavescens*, Po = *Porolithon onkodes*.

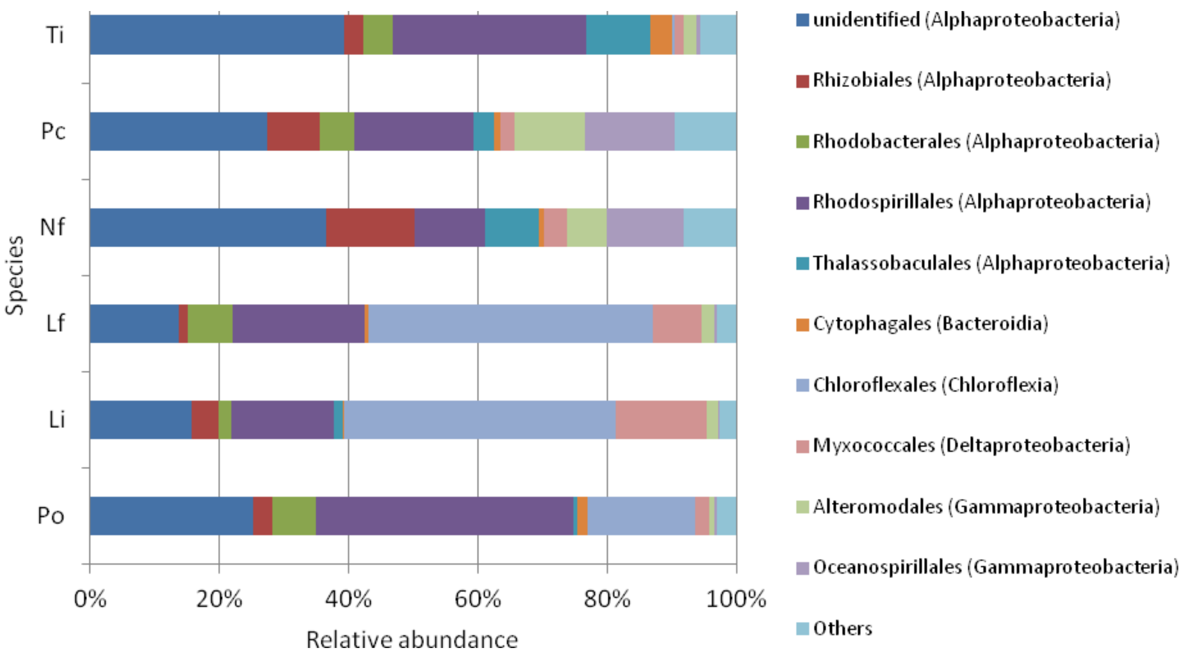

Supplementary figure S2: Relative abundance of ASVs at the order level for the different CCA species. Only the 9 most abundant orders are shown, with their class in parenthesis. Species abbreviation as in figure S1

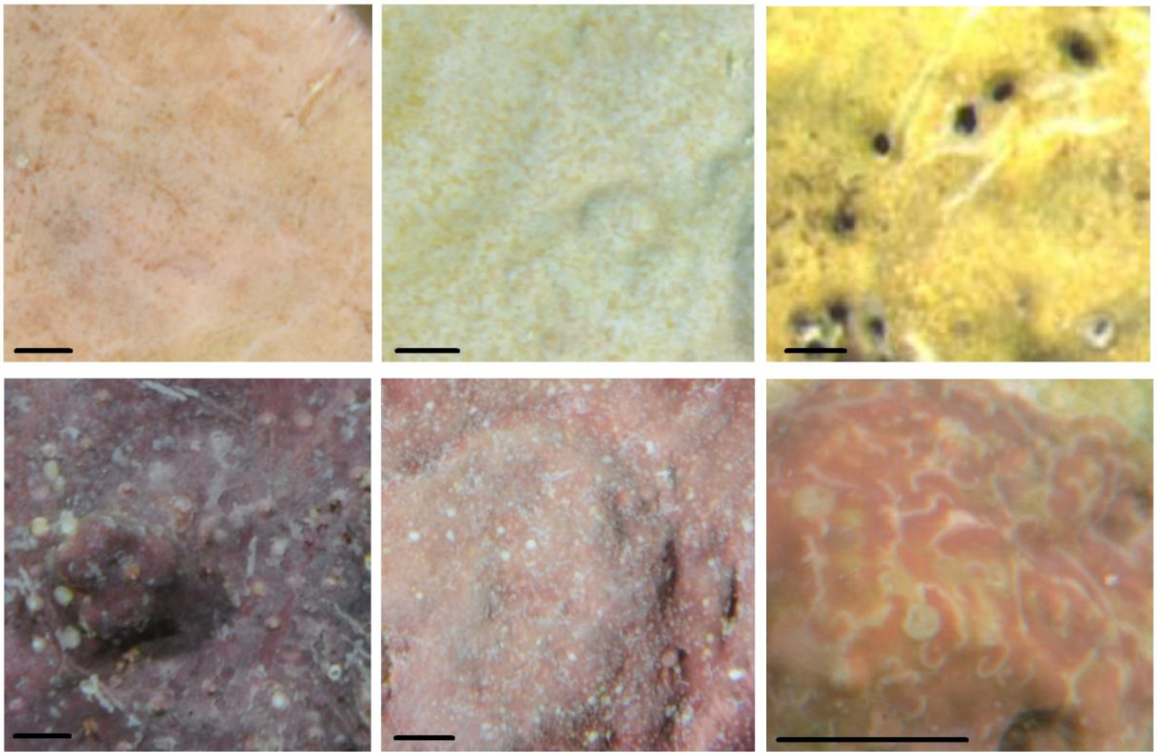

Supplementary figure S3: Close-ups of the different species of crustose coralline algae: a) *Porolithon onkodes* b) *Lithophyllum insipidum* c) *Lithophyllum flavescens* d) *Neogoniolithon fosliei* e) *Paragoniolithon conicum* f) *Titanoderma prototypum*. Scale bar = 5mm.
